# Psychometric Properties of the Connor-Davidson Resilience Scale for South America (CD-RISC-25^SA^) in Peruvian Adolescents

**DOI:** 10.3390/children9111689

**Published:** 2022-11-03

**Authors:** Karen A. Dominguez-Cancino, Francisca L. Calderon-Maldonado, Edith Choque-Medrano, Carola E. Bravo-Tare, Patrick A. Palmieri

**Affiliations:** 1 EBHC South America: A JBI Affiliated Group, Calle Cartavio 406, Lima 15023, Peru; 2 Escuela de Enfermería, Universidad Científica del Sur, Ctra. Panamericana S. 19, Villa EL Salvador 15067, Peru; 3 Addiction Study Program, Université de Sherbrooke, 150, Place Charles-Le Moyne, Bureau 200, Longueuil, QC J4K 0A8, Canada; 4 Department of Mathematics and Computer Science, Universidad Santiago de Chile, Av. Libertador Bernardo O’Higgins 3363, Santiago 9170022, Chile; 5 Escuela de Enfermería, Universidad Norbert Wiener, Av. Arequipa 444, Lima 15046, Peru; 6 South American Center for Qualitative Research, Universidad Norbert Wiener, Av. Arequipa 444, Lima 15046, Peru; 7 College of Graduate Health Studies, A. T. Still University, 800 West Jefferson Street, Kirksville, MO 63501, USA; 8 Center for Global Nursing, Texas Woman’s University, 6700 Fannin Street, Houston, TX 77030, USA

**Keywords:** adolescents, CD-RISC, Connor-Davidson Resilience Scale, cross-cultural research, factor analysis, Peru, psychometric, reliability, resilience, South America, translation, validity

## Abstract

Resilience describes the ability of someone to adapt to adverse life experiences by adjusting to demands with behavioral flexibility. When encountering crisis situations, resilient people typically spring back emotionally with increased strength and internal composure. Measuring resilience is important for assessing the ability of adolescents to respond to adverse situations. The objective of this study was to evaluate the psychometric performance of the Spanish version of the Connor-Davidson Resilience Scale (CD-RISC) © for South America (CD-RISC-25^SA^) in a population of vulnerable Peruvian adolescents. This study used a cross-sectional design to measure sociodemographic variables and resilience. Participants were 451 adolescents living in a shelter in Lima, Perú. Face and content validity were established by expert panel, construct validity was evaluated with exploratory and confirmatory factor analysis, and internal consistency was assessed with Cronbach’s alpha. The analysis resulted in a four-dimensional model with 22 items explaining almost 27% of the variance with a Cronbach’s alpha of 0.90. The dimensions included self-confidence and self-trust from previous experiences, internal resources to cope with difficult situations, personal competence and tenacity, and self-regulation with external resources. Two of the 3 items eliminated from the instrument were related to the original dimension “spirituality influences” which may have been incorrectly translated and adapted without equivalence of meaning for cross-cultural research. The CD-RISC-25^SA^ is not a stable multidimensional instrument for measuring resilience across the cultures and contexts of countries. However, the instrument appears to be stable for measuring resilience as a single dimension. For measuring resilience in the context of Peru, a four-dimensional model with 22 items was validated. Variations in the psychometric properties of translated instruments may result from not establishing the equivalence of meaning for each item before performing cross-cultural research. Researchers need to search for a more precise understanding of resilience as a universal concept transferable across borders and through translations.

## 1. Introduction

From a life course approach [1], the origin of diseases in advanced ages are explained by internal and external factors that include the effects of social determinants of health in critical and sensitive periods of human development, such as childhood, adolescence, and the early years of adult life [2]. Health status results from the interactions of protective factors with risk factors arising from various etiologies, including biological, psychological, behavioral, cultural, economic, social, and environmental factors [3,4]. These factors not only impact an individual but are also transmitted to subsequent generations and explain the cumulative effects of risk exposure and the mitigation by protective factors that become a pattern for health [2].

Adolescents living in institutional shelters are socially vulnerable with developmental risk factors resulting from inadequate social support [5]. A concept analysis of adolescents living in shelters [6] defined vulnerability as “the constellation of past, present and future risk, perceived or real, because of the common human experience of risk, the increased vulnerability of the adolescent period, the consequences of family disruption, and the increased risks of life on the street” (p. 2824). Without adequate family support in the context of vulnerability, adolescents need inner resources to cope with their situation and the resiliency to develop adaptive strategies to achieve a healthy self-identity [5,6,7]. In Peru, at least 19,000 children and adolescents are living in institutions for protective reasons [8]. Data is unavailable for the larger number of adolescents believed to be living in the streets or informal settings without their family [9]. This living situation may partially explain why 37% of Peruvian adolescents work more than 36 hours per week [10].

Vulnerability can be modified with interventions at critical points, facilitating harm prevention and promoting well-being through protective factors, such as resilience, that can counteract adverse effects arising from the environment [2]. In this sense, adolescence is a formative part of the life span because of the frequent biological changes and the gradual shift from childhood to adulthood. The process of “growing up” promotes adolescents in society as more autonomous people capable of choosing behaviors, habits, and lifestyles [2,11]. For adolescents, resilience can be described as the capacity for social flexibility [12], resulting in their ability to respond to adversity and to positively adapt [13]. For this reason, accurate resilience measurement is the precursor to designing interventions that facilitate the positive development of vulnerable adolescents.

## 2. Background

Resilience corresponds to a set of positive individual qualities that favor adaptation [14] and includes the ability to overcome the negative effects of risk factors, successfully cope with traumatic events, and avoid negative paths [15]. The original concept of resilience is closely attached to child developmental psychology [16], where some children were observed to reach adulthood less harmed by significant historical events and personal adversity than others. In high school students, for example, factors linked to resilience include confidence, coordination, control, composure, and commitment [17]. The possibility of adapting to and coping with adversity is diminished when exposed to chemical abuse, domestic violence, education failure, or family poverty [18]. As such, positive interventions to improve resilience of adolescents can reduce the number of risks encountered and build the protective factors that facilitate healthy development.

Resilience can be developed and determined by factors in social environments [19]. Adolescents with strong positive relationships with significant figures or who have religious affiliations and participate in social activities are more likely to overcome adversity [20]. For adolescents lacking a secure foundation with their immediate or extended family, cultivating a network or “base camp” of social support from work, social, educational, recreational, and professional relationships can provide a foundation for successfully managing adverse situations [18,21,22]. Institutionalization is one way to decrease risk factors by removing an adolescent from an unhealthy environment and providing them with the opportunity to cultivate their base camp through development of healthy relationships with peers, teachers, and caregivers [23].

### Connor-Davidson Resilience Scale

Resilience is an adaptative behavior more than an individual characteristic and is related to internal and external factors. Measuring resilience is important for mental health professionals and educators to be able to identify the capability of adolescents for responding to adverse situations in institutionalized environments. Additionally, measurement tools are important for assessing the outcomes of interventions intended to improve the health and well-being of vulnerable adolescents. Although there are at least 15 different instruments to measure resilience, the Connor-Davidson Resilience Scale (CD-RISC)© is the most widely used [24] and has the most consistent psychometric properties [25,26], but moderate psychometric quality [25]. The CD-RISC has largely been used in studies of adults [24], but some international studies in Australia [27], China [28], and South Africa [29] focused on adolescents.

There are at least five CD-RISC versions reported in the literature, including the original 25-item, five-dimensional CD-RISC-25 [30]; the 17-item, three-dimensional scale CD-RISC-17 [31]; and three one-dimensional scale versions represented by the 10-item CD-RISC-10 [32], 7-item CD-RISC-7 [33], and 2-item CD-RISC-2 [34]. Importantly, only three versions, the CD-RISC 25-item, 10-item, and 2-item scales, are authorized for research under the terms of the copyright [35]. Items are scored with a 5-point Likert scale ranging from 0 (not true at all) to 4 (true nearly all the time) points. The total score ranges from 0 points for the lowest resilience in all versions of the CD-RISC to the highest resilience which varies by scale: 100 (CD-RISC-25), 40 (CD-RISC-10), and 8 (CD-RISC-2) points [30].

A Spanish-language version of the CD-RISC-10 was reported from Spain [36] in a study of young adults and recently from Peru [37] in a study of adolescent mothers. These versions were noted to be authorized for research. However, another study reported use of an unauthorized 7-item CD-RISC in a population of college students in Peru [33]. Despite the availability of an authorized Spanish version of the CD-RISC-25 for South America, CD-RISC-25^SA^ [38], there were no studies identified that cited the application of the authorized instrument. As such, the purpose of this exploratory study was to determine the validity and reliability of the authorized Spanish language version of the CD-RISC-25^SA^ in a population of vulnerable adolescents in Peru.

## 3. Materials and Methods

### 3.1. Study Design

This study used a cross-sectional design for psychometric analysis. The results of this study are reported in accordance with the Strengthening the Reporting of Observational Studies in Epidemiology, STROBE, recommendations for cross-sectional studies [39].

### 3.2. Setting and Participants

The study was conducted at a residential shelter for children in Lima, Peru. The shelter, operated by a nongovernmental organization, provides residential, educational, and health services to vulnerable adolescents. Participants were recruited through convenience sampling of adolescents living at the residential shelter. The population at the shelter is transitory with approximately 500 adolescents receiving services. Early (10 to 13 years) and middle (14 to 17 years) age adolescents [40] living at the shelter for at least three months were eligible to participate. Recognizing the residential longevity of many residents, adolescents 18 years old (late age) living at the shelter for at least one year during middle adolescence were also eligible to participate. Exclusion criteria were adolescents unwilling to assent or parents or guardians unwilling to consent.

### 3.3. Data Collection

Data was collected in a classroom at the shelter using a 29-item instrument with sociodemographic section and the CD-RISC-25^SA^. Although the CD-RISC-25^SA^ requires 10 minutes to complete, the total participant time for ascent, consent, and completion of the CD-RISC was 45 minutes. The sociodemographic section was 4 items requesting information for age (10 to 18 years), gender (male or female), level of education or employment status (primary school, high school, or work), and residential modality (part-time or full-time residence at the shelter). Resilience was operationalized with the CD-RISC-25^SA^. Although participants completed a paper instrument, they were also offered the opportunity to complete the instrument verbally. The resulting data were organized in an Excel spreadsheet before statistical analysis.

#### Connor-Davidson Resilience Scale for South America

All versions of the CD-RISC are copyrighted, and some versions are translated into different languages. Since the copyright owner retains the exclusive rights and ability to authorize others to reproduce the copyrighted instrument and display the copyrighted instrument publicly [41], the CD-RISC-25^SA^ and the user manual were obtained directly from the authors for cross-cultural research in Peru. The instrument measures resilience through 25 items with five subscales that include the following: “positive acceptance of change and secure relationships” (5 items); “to trust in one´s instincts, tolerance of negative affect, and strengthening effects of stress” (7 items); “personal competence, high standards and tenacity” (8 items); “control” (3 items); and “spiritual influences” (2 items). The item summary with dimensions is provided in Table 1.

The item responses are recorded with a 5-point Likert scale with assigned points to calculate resilience. Possible responses include: "not true at all” (0 points), “rarely true” (1 point), “sometimes true” (2 points), “often true” (3 points), and “true nearly all the time” (4 points). The composite score ranges from least to most resilient (0 to 100 points).

### 3.4. Face and Content Validity

The authorized CD-RISC-25^SA^ is copyrighted, which prevents alteration without permission. Although the user manual states the Spanish translation for South America is psychometrically valid [35], face and content validity were established for the Peruvian context with an expert panel of five participants. Invitations were extended to clinical mental health professionals with experience working with vulnerable adolescents. The percentage of agreement for each of five characteristics—congruency, breadth of content, redaction, clarity and precision, and pertinence of the items—needed to be achieved with at least 80% for each item and 90% overall. An agreement of 90% or greater was achieved for each criterion and the total scale. Therefore, no changes were made to the instrument. 

### 3.5. Data Analysis

For data analysis, sample A (n = 451) was analyzed for the exploratory factor analysis. Then, sample B (n = 350) was created for confirmatory factor analysis using a simple random sample without replacement [42]. Descriptive statistics for sociodemographic variables were calculated for the description of the total sample and the created random sample. Multivariate normal distribution was verified by the Shapiro Wilk test; 5% significance of the distribution was not followed. The psychometric analysis was performed in four sequential stages [43]: (1) exploration of the data, (2) exploratory factor analysis considering the original structure of the instrument with the total sample, (3) confirmatory factor analysis of the theoretical structure with the random sample, and (4) confirmatory factor analysis with the random sample for the proposed structure. The procedure was completed to evaluate the original model and to test a proposed new model, as necessary [44,45]. All the analyses were performed in statistical software R version 3.6.1 [46].

#### 3.5.1. Exploratory Factor Analysis 

The exploratory factor analysis was completed with the maximum likelihood method [47,48,49]. Factorial utilization and sample adequacy were evaluated with the Bartlett’s and Kaiser-Meyer-Olkin tests of sphericity [50]. For factor identification, polychoric correlation was used, considering the nature of the instrument with a 5-point Likert scale scored from 0 to 4 points, and an Oblimin rotation was applied [50,51,52]. To complement the construct validation, the Spearman correlation was used between each item to the factor and each factor to the test. To evaluate instrument reliability, or internal consistency, the Cronbach alpha [53] was calculated using polychoric correlation matrices [54].

#### 3.5.2. Confirmatory Factor Analysis 

Confirmatory factor analysis was completed with the maximum likelihood method [48,55], including the following goodness of fit statistics: *X^2^/df*, standardized mean square residuals, comparative fit index, Tucker-Lewis index, and standardized root mean square residues. Standardized mean square residuals less than 0.08, values of the comparative fit index and Tucker-Lewis index greater than 0.90, and standardized root mean square residues less than 0.05 were considered as acceptability criteria for the analysis [56]. 

### 3.6. Ethical Considerations

The study protocol (ID-006-17) was approved by the university ethics committee in Peru and conducted according to the guidelines provided by the Declaration of Helsinki [57]. Acknowledging their emerging autonomy [58], assent was obtained from each adolescent [59], following a recommended age-specific operational definition [60]. Obtaining adolescent assent before parental informed consent is important in developing countries to protect the human rights of adolescents [61]. As such, informed consent was sought from a parent or guardian after assent was provided by the adolescent.

## 4. Results

The analysis included 451 participants. The mean age was 14 years (range 11 to 18 years), half were female, almost all were in high school, and most were not permanent shelter residents. The sociodemographic characteristics of participants are presented in Table 2. There were no significant differences between sample groups A and B.

### 4.1. Exploratory Factor Analysis

**Figure 1:**
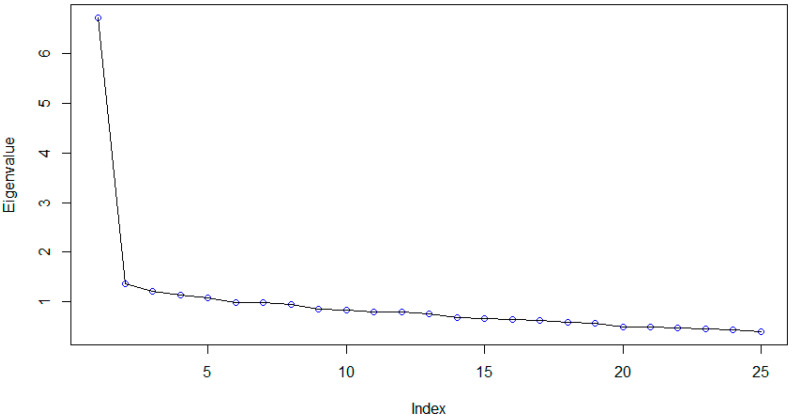
Scree Plot of the Exploratory Factor Analysis for the General Sample.

Exploratory factor analyses were performed with the total sample. First, we analyzed the adequacy of the procedure using sphericity tests, obtaining a value of 0.88 for the Kaiser-Meyer-Olkin test and *p* < 0.001 for Bartlett’s test. For the original CD-RISC-25^SA^, the overall Cronbach alpha was very good at 0.90. We kept all the factors to analyze the original five-dimensional structure of the instrument. The scree plot provided in Figure 1 indicates the unidimensional nature of the CD-RISC-25^SA^.

The mean values for each item, inter-test correlation, factor loading, and Cronbach alpha of each dimension are presented in Table 3. Although we respected the original five-dimension CD-RISC-25^SA^ model, analysis indicated the presence of a four-dimensional model with a different item distribution. Using a factor loading cut point of 0.2 as reference [62], we determined it is appropriate to eliminate items 2 and 3. Item 3 correlated in a negative way with the fifth dimension, leaving it with one item that also should be removed. The eliminated items are bold in the table. The new model, with the eliminated items, explained 24.3% of the construct. The Cronbach alpha measurement for each dimension indicated poor internal consistency.

### 4.2. Confirmatory Factor Analysis

The original five-factor model proposed by Connor and Davidson [30] was tested with a simple random sample as presented in Table 4.

**Figure 2:**
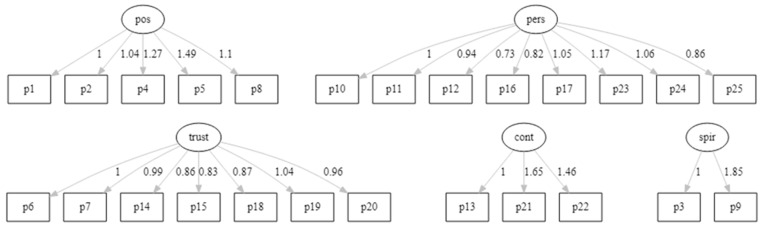
Confirmatory Analysis Path Diagram for the Original Five-Factor Model. Abbreviations: cont, control; pers, personal competence, high standards and tenacity; pos, positive acceptance of change and secure relationships; spir, spiritual influences; trust, trust in one´s instincts, tolerance of negative affect, and strengthening effects of stress.

The confirmatory factor analysis path diagram for the five-factor model proposed by Connor and Davidson, with standardized factor loadings, is presented in Figure 2.

The adjusted measurement was acceptable but outside the cut point for good fit [63,64]. Considering the results from exploratory factor analysis [62], a new confirmatory factor analysis was performed with the simple random sample for the four-dimensional model, excluding items 1, 2, and 3. The proposed four-dimensional model, with new item distribution, provided acceptable results. The model explained the concept of resilience with 22-items in four dimensions (Table 5): self-confidence and self-trust from previous experiences, internal resources to cope with difficulties, personal competence and tenacity, and self-regulation and external resources.

**Figure 3:**
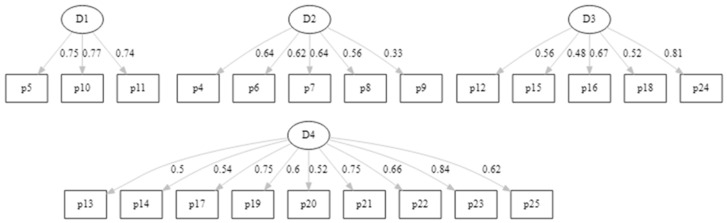
Confirmatory Analysis Path Diagram for the Four-Factor Model*. *D1 self-confidence and self-trust from previous experiences, D2 internal resources to cope with difficulties, D3 personal competence and tenacity, and D4 self-regulation and external resources.

In the proposed model, the dimension 1 aligned with self-confidence in a wide perspective, while dimensions 2, 3, and 4 were related to the specific resources that help adolescents be resilient. The confirmatory factor analysis path diagram for the proposed four-factor model for resilience is presented in Figure 3.

The results presented in Table 6 confirmed this alignment because dimension 1 had the highest statistically significant correlation with the other three dimensions. 

In the case of dimension 2, a weak yet still significant association was observed with dimensions 3 and 4. This finding indicated the internal resources to cope with difficulties were associated with tenacity and emotional self-regulation. As such, tenacity implies the existence of emotional self-regulation. These findings can be explained by the one-dimensional model presented in the previous scree plot presented in Figure 1.

## 5. Discussion

The purpose of this study was to evaluate the content validity, construct validity, and reliability of the CD-RISC-25^SA^ in a population of vulnerable adolescents living in a shelter in Peru. The result of our analyses was a four-dimensional, 22-item model explaining 26.4% of the concept variance with a 0.90 Cronbach alpha. The new model’s four dimensions included self-confidence and self-trust from previous experiences, internal resources to cope with difficulties, personal competence and tenacity, and self-regulation and external resources. Three items were eliminated from the instrument, possibly because of poor item translation and adaptation from English to Spanish.

### 5.1. Four-Dimensional Model

The four-dimensional model of this study aligns with the common domains of resilience reported by Latino immigrant families in the United States, such as individual characteristics, family strengths, cultural factors, and community supports [65]. The first dimension was related to the confidence of the adolescent using their resources that were reaffirmed by personal history. The second dimension reflected the personal resources used to cope with difficult situations, such as strength, humor, and logical thinking. The third dimension was related to the internal resources that help adolescents continue forward on their path no matter the obstacles. The final dimension included the components of emotional self-regulation and support systems that connect the adolescent to the world by providing a sense of purpose and someone to go to for help. Comparing the factors from the original structure of the instrument, the dimension “personal competence, high standards and tenacity” corresponded to the dimension that represented 32% of the items in the original instrument, “positive acceptance of change and secure relationships”, with a redistribution of the items to three of four new dimensions. Control was the only dimension remaining intact from the original instrument, but it emerged as part of a new dimension, self-regulation and external resources.

Our findings partially align with the few studies of adolescent Latinos reported from the United States. Some studies applied the 10-item rather than 25-item CD-RISC with good results. Importantly, the 10-item version does not have two of the three items eliminated from the new model proposed in this study. These items focus on the individual resources of people without considering external elements [36,66,67,68,69]. There are few studies from Latin America that apply the recommended 25-item CD-RISC and keep all items before principal component or structural equation analysis [70,71]. Most often, the spiritual items are missing from the instrument [72,73,74]. Only one study with participants from Spain and Chile reportedly maintained the original five dimensions [70].

Performance variability of the CD-RISC has also been observed in populations other than Latinos. In a critical review of scales used for resilience research [26], the CD-RISC-25 was observed to have multiple refinements, validations, and revalidations, resulting in different factor arrangements that included five, four, three, two, and one factor models. When compared with 20 other instruments that measure the same construct, Salisu and Hashim [26] reported the CD-RISC-25 is the most dominant scale for assessing resilience because of its more consistent psychometric properties. However, some researchers have suggested the CD-RISC-10 as a unidimensional model is more stable across cultures and contexts [32,75]. For this reason, more research is necessary to understand the item level dynamics resulting in the different factor models.

### 5.2. Items Eliminated

In the case of this study, the three items eliminated for the new version of the scale were “able to adapt to change”, “close and secure relationships”, and “sometimes fate or God can help me”. Although resilience has been defined in different ways, most researchers agree resilience is the ability of individuals to adapt, thrive, cope, or bounce back from adversity with a strengthened self at the end of a crisis [26]. Similarly, there is agreement that resilience differs by culture, context, or condition, such as the nature of threats or the type of event [26,76,77,78,79]. Since vulnerable adolescents living in a developing country, such as Peru, often deal with serious problems, confront life changing events, and experience social injustice in the absence of government assistance programs, adaptation may be a normalized approach to daily living. As a result, adverse conditions become common rather than special cause. This may be a plausible explanation for the elimination of the “ability to adapt to change, a distinct characteristic of resilience”, from the model in this study. For a deeper understanding of this finding, qualitative research should be undertaken to determine the role of resilience in the lived experiences of vulnerable adolescents. 

Regarding the item about close and secure relationships, vulnerable adolescents living in a shelter may sometimes have more external mobility. This means these adolescents engage in more interactions with people at school, work, and possibly their homes. Although more opportunities to build close and secure relationships in the protective shelter environment is one explanation for the item about close and secure relationships being eliminated from the model, the broader literature indicates building secure attachments is an important mediator for developing resiliency [18,80]. Institutionalized adolescents usually have problems developing secure attachments without their families and difficulties establishing attachments with adults at the institution [22,23]. As such, relationship instability may explain why close and secure relationships is not as important for participants in this study as in other studies. 

Finally, the items related to fate and God may have been eliminated from the model because of poor translation of items from English to Spanish. When retrospectively analyzing the CD-RISC-25 instrument, we observed item meanings may not have been conveyed for countries in South America. For example, the item “sometimes luck or God can help me when there are no clear solutions to my problems” [73] included in the CD-RISC-25^SA^ is remarkably different from “sometimes fate or God can help me” [30] in the CD-RISC-25. In South America, luck is usually related to chance, conceptually more like a gamble, whereas fate is related to destiny with a religious connotation or spiritual explanation. For this reason, the items related to spirituality may need to be translated and re-validated through cognitive interviews with bilingual experts to make sure the same question is asked in the same manner with the same intended meaning as the source item [81].

### 5.3. Resilience in the Context of South America

Considering our findings related to the four-dimensional model and previously described item eliminations, researchers need to continue searching for a more precise understanding of resilience within the cultures and contexts of South American countries. These countries have unique cultural characteristics related to family structures, social interactions, community values, and the Catholic religion that represent important sources of strength when coping with adverse situations and difficult life experiences [82,83,84]. The social dynamics for vulnerable adolescents living in developing countries, such as Peru, is also another factor for additional consideration because of social injustices and economic inequalities. The distinct differences in the culture and economic contexts of developing countries in South America is largely unrecognized in the literature.

Psychometric studies reported by researchers in Sweden and Spain highlight the importance of further exploration within the context of South America. Swedish researchers observed the spirituality dimension was dropped from their model because less than a third of their population identify as religious [85]. Similarly, Spanish researchers reported the spirituality dimension was dropped from their models [74,86]. Even though 62% of people in the world define themselves as religious [87], few Spanish people identify as religious (37%), and the majority identify as not religious (35%) or atheist (20%) [88,89]. Yet, most Peruvians identify as religious (82%), and very few identify as not religious (11%) or atheist (2%). This data may explain psychometric differences in the psychometric performance of the CD-RISC-25 between Spain and other Spanish-language contexts in South America. For this reason, researchers need to recognize the distinct variations in the context of target populations, which can alter the performance of a multidimensional instrument, such as the CD-RISC-25, before using it for cross-cultural research. For this reason, the psychometric performance of specific dimensions should be scrutinized when variations in results at the dimension level conflict with other studies. As such, instruments translated from English into Spanish and validated for use in Spain should not be assumed to be transferable to the culture and context of other Spanish speaking countries in Latin America, such as Peru. 

From a methodological perspective, the original dimension “spirituality influences” may have two problems that need to be reconciled with additional research. First, the equivalence of the item translations needs to be established for each country where the instrument has been translated for cross-cultural research. Second, if the items representing spirituality are not sensible for Peruvians, a dimension capable of explaining additional variance may be lost because of inadequate translation and adaptation. The loss of this dimension is an important observation since at least three items are needed to create a dimension for factor analysis procedures [50].

Because the proposed four-dimensional model for Peru explains a little more than a quarter of the variance for the concept of resilience, other factors need to be explored to increase the understanding of resilience in the context of Peru, and probably across South America as well. Increased understanding about resilience in the Peruvian context is important for promoting interventions with protective factors to prevent health risks associated with low resilience [90] and to support adolescents as they develop the capacity to confront normative and nonnormative crises [91]. Identifying other factors for resilience may also result in the development of more effective interventions to increase adolescent well-being [18,22,23].

### 5.4. Limitations

This study has some limitations. First, we identified potential problems with the transcultural translation, adaptation, and validation of the instrument in the advanced stages of a longitudinal research project. Although we would expect to capture the issues described in this study during the face and content validity stages of the project, that was not the case. Notably, the instructions accompanying the instrument indicate the CD-RISC-25^SA^ is valid and reliable for countries in South America. For these reasons, we highly recommend including translation and linguistics experts during the process to establish face validity, inviting members of the target population during the process to establish content validity, and conducting a small pilot study with the target population to test the instrument before advancing a project. Nonetheless, this limitation is also an important finding to be considered for future research using translated instruments. 

Spirituality, as well as religion, has a central role in South American communities. Most of the population in South America countries identify with the Catholic religion. As such, measurement instruments containing constructs related to spirituality, religion, and faith are aligned with the population. Since the Catholic religion is an important foundation for Peruvian culture, it is embedded in the structures and functions of families, communities, organizations, and government. Therefore, the spirituality dimension should be expanded with more items to improve the psychometric performance. 

Although the study was powered to complete one phase of psychometric analysis, an additional random sample was created to permit confirmatory factor analysis. However, the methods adhered to established recommendations for psychometric analysis [48,92]. Although our rigorous study design incorporated methods to minimize selection bias, response bias, information bias, social desirability bias, and nonprobability sampling bias, these are normal limitations for instrument studies [93].

## 6. Conclusions

The CD-RISC-25^SA^ may not be a valid and reliable multidimensional instrument for measuring resilience across cultures, contexts, and countries in South America. However, the CD-RISC-25^SA^ seems to be a stable unidimensional measurement for resilience. In the context of Peru, the CD-RISC-25^SA^ was valid and reliable for measuring resilience with a four-dimensional, 22-item scale. Additional research is needed to understand the concept of resilience in the context of Latin America represented by the original five dimensions of the CD-RISC-25. Differences in the religious context of countries and poor instrument translations that failed to achieve equivalence of meaning for each item seemed to negatively impact dimension performance. In particular, the items associated with the spiritual influences dimension need to be improved for the CD-RISC-25^SA^. More research is also necessary to establish construct and convergent validity of Spanish-language versions of instruments for measuring resilience. Translated versions of the CD-RISC-25 should be assessed not only for validity but also for equivalence of meaning before use in cross-cultural research.

## Figures and Tables

**Table 1:** Item Summary with Dimensions of the CD-RISC-25^SA^.

Item No.	Item Description	Dimension with Description	Items by Dimension
1	Able to adapt to change	Dimension 1: Positive acceptance of change and secure relationships	1, 2, 4, 5, 8
2	Close and secure relationships		
3	Sometimes fate or God can help me		
4	Can deal with whatever comes		
5	Past success gives confidence for new challenge		
6	See the humorous side of things	Dimension 2: Trust in one´s instincts, tolerance of negative affect, and strengthening effects of stress	6, 7, 14, 15, 18, 19, 20
7	Coping with stress strengthens		
8	Tend to bounce back after illness or hardship		
9	Things happen for a reason		
10	Best effort no matter what		
11	You can achieve your goals		
12	Things look hopeless, I don’t give up		
13	Know where to turn for help	Dimension 3: Personal competence, high standards and tenacity	10, 11, 12, 16, 17, 23, 24, 25
14	Under pressure, focus and think clearly		
15	Prefer to take lead in problem solving		
16	Not easily discouraged by failure		
17	Think of self as strong person		
18	Make unpopular or difficult decisions		
19	Can handle unpleasant feelings		
20	Have to act on a hunch		
21	Strong sense of purpose	Dimension 4: Control	13, 21, 22
22	In control of your life		
23	I like challenges		
24	You work to attain your goals	Dimension 5: Spiritual influences	3, 9
25	Pride in your achievements		

This table was adapted from Connor and Davison [30] and Davidson [35].

**Table 2:** Sociodemographic Characteristics of Samples A and B.

Variables	Sample A (n = 451)	Sample B (n = 350)
Age (X, min-max)	13.74 [11,12,13,14,15,16,17,18]	13.69 [11,12,13,14,15,16,17,18]
Gender (n, %)		
Male	219 (48.6)	158 (45.1)
Female	232 (51.4)	192 (54.9)
Level (n, %)		
Primary school	48 (10.6)	35 (10.0)
High school	389 (86.3)	305 (87.1)
Working or other academic condition	14 (3.1)	10 (2.9)
Modality (n, %)		
Internal Presence	160 (35.5)	126 (36.0)
External Presence	291 (64.5)	224 (64.0)

**Table 3:** Summary Data of Items, Inter-Test Correlation, and Factor Loading.

Item No.	Item Description	Mean	Item-Test	Factor 1	Factor 2	Factor 3	Factor 4	Factor 5
21	Strong sense of purpose	2.77	0.57	0.652				
22	In control of your life	2.51	0.53	0.531				
25	Pride in your achievements	3.16	0.56	0.437				
14	Under pressure, focus and think clearly	2.24	0.51	0.371				
23	I like challenges	2.54	0.66	0.318				
17	Think of self as strong person	2.61	0.64	0.301				
20	Have to act on a hunch	2.19	0.50	0.253				
19	Can handle unpleasant feelings	2.23	0.53	0.236				
13	Know where to turn for help	2.39	0.46	0.225				
16	Not easily discouraged by failure	2.28	0.54		0.673			
12	When things look hopeless, I don’t give up	2.44	0.42		0.484			
18	Make unpopular or difficult decisions	1.93	0.46		0.313			
15	Prefer to take the lead in problem solving	2.48	0.50		0.311			
24	You work to attain your goals	2.50	0.58		0.243			
2	**Close and secure relationships**	2.05	0.39		**0.190**			
6	See the humorous side of things	2.36	0.46			0.655		
7	Coping with stress strengthens	2.54	0.55			0.417		
9	Things happen for a reason	2.51	0.35			0.292		
8	Tend to bounce back after illness or hardship	2.48	0.48			0.226		
4	Can deal with whatever comes	2.37	0.57			0.217		
10	Best effort no matter what	2.81	0.60				0.626	
11	You can achieve your goals	2.84	0.61				0.538	
5	Past success gives confidence for new challenges	2.76	0.57				0.368	
3	**Sometimes fate or God can help me**	2.20	0.27					**−0.394**
1	**Able to adapt to change**	2.47	0.47					**0.289**
Eigen values				6.73	1.39	1.22	1.13	1.08
Variance explained (%)				6.3%	5.4%	5%	5%	2.6%
Cronbach alpha				0.56	0.68	0.80	0.53	0.27

**Table 4:** Model Indexes for Sample B.

Adjusted Model Index for Sample B	Five Dimension Model	Four Dimension Model
*X^2^/df*	<0.0001	<0.0001
Root Mean Squared Error of Approximation (RMSEA)	0.043	0.041
Comparative Fit Index (CFI)	0.899	0.926
Tucker Lewis Index (TLI)	0.886	0.916
Standardized Root Mean Square Residues (SRMR)	0.050	0.047

**Table 5:** Proposed Model for Resilience with Dimensions and Items.

Item No.	Item Description	Proposed Dimension with Description
5	Past success gives confidence for new challenges	Dimension 1: Self-confidence and self-trust from previous experiences
10	Best effort no matter what	
11	You can achieve your goals	
4	Can deal with whatever comes	Dimension 2: Internal resources to cope with difficulties
6	See the humorous side of things	
7	Coping with stress strengthens	
8	Tend to bounce back after illness or hardship	
9	Things happen for a reason	
12	When things look hopeless, I don’t give up	Dimension 3: Personal competency and tenacity
15	Prefer to take the lead in problem solving	
16	Not easily discouraged by failure	
18	Make unpopular or difficult decisions	
24	You work to attain your goals	
13	Know where to turn for help	Dimension 4: Self-regulation and external resources
14	Under pressure, focus and think clearly	
17	Think of self as strong person	
19	Can handle unpleasant feelings	
20	Have to act on a hunch	
21	Strong sense of purpose	
22	In control of your life	
23	I like challenges	
25	Pride in your achievements	

**Table 6:** Covariance Among New Dimensions.

Main Dimension	Related Dimension	Covariance	Standard Error	*p*-Value
Dimension 1	Dimension 2 Dimension 3 Dimension 4	0.413 0.316 0.311	0.062 0.056 0.056	<0.0001 <0.0001 <0.0001
Dimension 2	Dimension 3 Dimension 4	0.288 0.253	0.054 0.049	<0.0001 <0.0001
Dimension 3	Dimension 4	0.244	0.050	<0.0001

## Data Availability

The aggregated data is available from the corresponding author on reasonable request.
